# Assessing sleep habits in school-aged children with phenylketonuria: a comparative study

**DOI:** 10.1007/s00431-025-06048-1

**Published:** 2025-03-05

**Authors:** Ayşe Mete Yeşil, Halil Tuna Akar, Yılmaz Yıldız, Serap Sivri, Elif N. Özmert

**Affiliations:** 1 Division of Developmental Pediatrics, Department of Pediatrics, Ankara Bilkent City Hospital, Ankara, Turkey; 2 Division of Developmental Pediatrics, Department of Pediatrics, Faculty of Medicine, Hacettepe University, Ankara, Turkey; 3 Division of Pediatric Metabolism, Department of Pediatrics, Faculty of Medicine, Hacettepe University, Ankara, Turkey; 4 Division of Pediatric Metabolic Disorders, Department of Pediatrics, Etlik City Hospital, Ankara, Turkey

**Keywords:** Phenylketonuria, Hyperphenylalaninemia, Sleep, CSHQ

## Abstract

Phenylketonuria (PKU) is an autosomal recessive metabolic disorder characterized as deficiencies in phenylalanine hydroxylase, leading to neurotoxic effects and neurodevelopmental challenges. Sleep, crucial for cognitive and behavioral development, remains underexplored in PKU populations. This study evaluates sleep characteristics and influencing factors in school-aged children with PKU compared to those with hyperphenylalaninemia (HPA) and healthy controls. A total of 101 children aged 5–10 years participated: 37 with PKU, 31 with HPA, and 33 healthy controls. Sleep quality and disturbances were assessed using the Children’s Sleep Habits Questionnaire (CSHQ) and parent-reported data. Additional factors, including phenylalanine levels and sleep hygiene practices, were analyzed. Despite no significant differences in total CSHQ scores across groups, children with PKU exhibited unique patterns, such as being less likely to awaken by himself or herself. Phenylalanine levels showed no significant correlations with overall sleep characteristics, except for an association with reduced sleep anxiety. Distinct sleep hygiene influences emerged in the HPA and control groups, while no such relationships were observed in PKU. This study underscores the complexity of sleep disturbances in PKU, highlighting the need for future research integrating biological, behavioral, and environmental factors. Identifying determinants of sleep problems will aid in developing tailored interventions to enhance the quality of life for patients with PKU.

**Table:** 

**What is known:** *• Children with PKU, in addition to the risks associated with chronic illness, may be more susceptible to sleep disturbances due to metabolic and neurochemical imbalances.* *• Prior studies on PKU and sleep have mainly focused on adult populations, with limited data available for school-aged children.*	
**What is new:** *• The sleep characteristics of PKU, HPA, and healthy controls were generally similar, except for awakening by themselves.* *• Distinct sleep hygiene influences were identified in the HPA and control groups, while no such associations were observed in the PKU group, highlighting the need for a broader perspective to explore alternative biological, behavioral, and environmental factors affecting sleep regulation in PKU.*

**Supplementary Information:**

The online version contains supplementary material available at 10.1007/s00431-025-06048-1.

## Introduction

Phenylketonuria (PKU) is an autosomal recessive metabolic disorder caused phenylalanine hydroxylase deficiency, leading to elevated phenylalanine levels and associated neurotoxicity through mechanisms like demyelination, decreased neurotransmitter production, and impaired neuronal survival. Untreated PKU results in developmental delays, intellectual disabilities, seizures, and psychiatric symptoms [1–3].

In healthy individuals, Phe levels are < 120 μmol/L. Individuals with mild hyperphenylalaninemia (untreated Phe 120–360 μmol/L, hereby referred as hyperphenylalaninemia, HPA) are not considered to be at risk of neuropsychiatric impairment [4–7]. It is universally accepted that Phe levels exceeding 360 μmol/L in individuals younger than 12 years should be treated and Phe levels maintained in the target range of 120–360 μmol/L [1–3]. Treatment is life-long, but target “Phe level ranges for individuals older than 12 years are varied. While different classification schemes exist for phenylalanine hydroxylase deficiency, in this paper we use the term “PKU” for all individuals with treatment-requiring phenylalanine hydroxylase deficiency (untreated Phe > 360 μmol/L), and recognize that in “classical PKU,” the most severe form of the disease, untreated Phe levels exceed 1200 μmol/L [4, 8–10].

Individuals with PKU are often treated with a Phe-restricted diet, which requires a significant reduction in the intake of natural protein sources. Instead, protein needs are met through the consumption of specially formulated Phe-free amino acid mixtures [4, 8–10]. Some individuals with PKU can be treated with sapropterin dihydrochloride, which provides high doses of the cofactor of the phenylalanine hydroxylase enzyme, which can reduce or eliminate the need for dietary treatment [4, 8–10]. The prognosis of PKU is shaped by the early initiation of treatment, the maintenance of optimal blood Phe levels, and the extent to which blood Phe is controlled over time [1, 3, 11]. While studies indicate that early and well-treated adults with PKU often achieve intelligence quotients (IQs) comparable to their unaffected family members, attention-related problems and executive functioning deficits remain areas of concern [12–14]. Poor dietary control in infancy has been associated with hyperactivity, temper tantrums, increased anxiety, and social withdrawal. Additionally, well-treated individuals may still face challenges such as depressive symptoms and low self-esteem, which are also observed in other chronic disease populations [13, 15, 16].

Given the neurodevelopmental and behavioral risks of PKU, sleep is a critical yet understudied factor in these patients, especially younger populations. Sleep is essential for cognitive and emotional functions, and inadequate sleep is linked to behavioral issues and impaired neurocognitive development in children [17, 18]. The chronic nature of PKU, including disruptions in neurotransmitter synthesis, suggests vulnerability to sleep disorders. Limited studies show varied outcomes; Bruinenberg et al.’s 2017 study found that adult patients experience poor sleep quality, increased latency, and daytime sleepiness. Animal models also showed altered activity patterns, supporting the hypothesis that PKU impacts sleep quality [19]. However, in contrast, another study involving early-treated patients with PKU (mean age 12 years) found that, despite deficits in melatonin and serotonin, the prevalence of sleep disorders was comparable to that of the general population [20]. A recent European survey on management practices highlighted that clinical symptoms, including sleep problems, are common among patients with PKU but are not systematically evaluated. This underscores the need for greater clinical attention to these issues [21].

Addressing sleep disturbances in patients with PKU is crucial for enhancing neurological and behavioral outcomes. This study investigates sleep patterns in school-aged children with PKU and compares them to those of children with HPA and healthy controls to better understand the unique sleep challenges faced by this population.

## Materials and methods

### Study population

The study population consisted of patients aged 60 to 119 months, diagnosed with PKU or HPA being followed at Hacettepe University Department of Pediatrics Metabolism Clinic. Exclusion criteria included patients with tetrahydrobiopterin deficiency, late-diagnosed PKU (treatment initiation age > 3 months), or cases with other chronic diseases. The control group consisted of healthy children of the same age range and gender, without any chronic diseases, recruited during *general pediatric outpatient clinic admissions.*All eligible patients presenting to the clinic during the study period (February 2022-February 2023) who met the inclusion criteria and consented to participate were included.

The study was approved by the ethics committee of Hacettepe University (GO22/233), in accordance with the declaraiton of Helsinki. Clinical data related to PKU/HPA were obtained from the patient records. Demographic and sleep hygiene-related data were collected through a questionnaire. The study was approved by the ethics committee of Hacettepe University (GO22/233), in accordance with the Declaration of Helsinki. Clinical data related to PKU/HPA were obtained from patient records, while demographic and sleep hygiene-related data were collected through a questionnaire. To assess children’s sleep quality and problems, the Children’s Sleep Habits Questionnaire (CSHQ) was utilized. The questionnaires were administered in person during outpatient clinic visits. Parents of the children were asked to fill out the forms face-to-face under the guidance of a healthcare professional to ensure accuracy and completeness. The CSHQ is an internationally recognized, 33-item tool originally developed by Owens et al. and validated for Turkish use by Perdahlı Fiş et al. It evaluates eight dimensions: bedtime resistance, sleep onset delay, sleep duration, sleep anxiety, night waking, parasomnia, sleep-disordered breathing, and daytime sleepiness. Parents complete the questionnaire retrospectively, recalling their child’s sleep habits over the previous week. Responses are generally rated as rarely (0–1 times/week), sometimes (2–4 times/week), or usually (5–7 times/week) ans scored as 1, 2, or 3 points, respectively. To ensure that higher scores consistently indicate more disturbed sleep, items 1, 2, 3, 10, 11, and 26 are reverse scored. A total score of 41 or above is the cut-off for clinically significant sleep problems [22, 23].

All collected data were uploaded to the SPSS statistical software for analysis. Due to some parents not answering all parameters, only those who provided responses were included in the relevant analyses. Categorical variables were analyzed using Pearson’s Chi-Square Test and Fisher’s Exact Test. The normality of numerical variables was assessed using visual (histograms, probability plots) and analytical methods (Kolmogorov–Smirnov/Shapiro–Wilk Test). Normally distributed variables were compared using the independent sample t-test and one-way ANOVA for two and three-group analyses, respectively. For non-normally distributed variables, Mann–Whitney U Test and Kruskal–Wallis Test were used for two and three-group comparisons, respectively, with Bonferroni correction applied to significant results. A *p*-value < 0.05 was considered statistically significant.

## Results

The study included a total of 101 participants: 37 with PKU, 31 with HPA, and 33 healthy controls. The age at diagnosis for patients with PKU ranged from 2 to 75 days, with a median of 13 days. Among the 31 patients diagnosed with HPA, all were managed without any special dietary or pharmacological interventions. Of the 37 patients diagnosed with phenylketonuria, 11 (29.37%) were receiving sapropterin dihydrochloride at therapeutic doses. Seven patients (18.92%) were monitored under a regimen of natural protein restriction alone, while 19 patients (51.35%) adhered to both natural protein restriction and supplementation of Phe-free amino acid formula. No use of melatonin, magnesium, or other sleep aids was reported among the children included in the study Among the 19 patients receiving the Phe-free amino acid formula, 14 had classical PKU, while 5 had milder forms of PKU. The sociodemographic characteristics of the study participants revealed no significant differences between the PKU, HPA, and healthy control groups in terms of age, gender distribution, birth order, and parental age (*p* > 0.05) (Table 1).

**Table 1:** Sociodemographic characteristics of the participants

Variable, *n* (%), mean (SD) or median (IQR)	PKU *n* = 37	HPA *n* = 31	Healthy control *n* = 33	Total *n* = 101	*p*
Age (years)	9 (7–10)	8 (7–10)	9 (7–10)	8 (7–10)	0.68
Gender					
Girls	21 (56.8%)	18 (58.1%)	19 (57.6%)	58 (57.4%)	1
Boys	16 (43.2%)	13 (41.9%)	14 (42.4%)	43 (43.6%)	
Maternal education					
< high school	28 (75.7%)	18 (58.1%)	19 (57.6%)	65 (64.4%)	0.19
≥ high school	9 (24.3%)	13(41.9%)	14 (42.4%)	36 (35.6%)	
Paternal education					
< high school	23 (62.2%)	20 (64.5%)	19 (57.6%)	62 (61.4%)	0.84
≥ high school	14 (37.8%)	11 (35.5%)	14 (42.4%)	39 (38.6%)	
Birth order					
First	20 (54.1%)	10 (32.3%)	14 (42.4%)	44 (43.6%)	0.19
Other	17 (45.9%)	21 (67.7%)	19 (57.6%)	57 (56.4%)	

*PKU* Phenylketonuria, *HPA* Hyperphenylalaninemia

Attention was also directed to sleep hygiene-related factors, which may play a critical role in understanding sleep outcomes across these groups. The median total screen time was 180 min (97.5–180). The median time for stopping physical activity before sleep was 60 min (30–90), the median time for stopping food intake was 90 min (30–120), and the median time for turning off screens before sleep time was 60 min (30–60). These sleep hygiene-related factors revealed no significant difference between the groups (*p* > 0.05). Reflux findings were reported in only 6 (5.9%) children in the whole group, and there was no significant difference between the study groups (*p* > 0.05).

Children’s Sleep Habits Questionnaire was utilized to compare sleep characteristics across the PKU, HPA, and control groups. Table 2 compares CSHQ scores across the three groups. No significant differences were found in median total CSHQ among the groups (*p* > 0.05). However, children in the PKU group were significantly less likely to be awakened by himself/herself (*p* < 0.001). Among the participating children, 56 (55.4%) had a clinically significant sleep risk score, with no significant difference in prevalence across the PKU (60.5%), HPA (53.3%), and control groups (51.5%) (*p* = 0.72).

**Table 2:** Comparison of the children’s sleep habits questionnaire scores and sleep patterns among PKU, HPA, and healthy control groups

Variable, *n* (%), or median (IQR)	PKU *n* = 37	HFA *n* = 31	Healthy control *n* = 33	Total *n* = 101	*p*
Total sleep duration (hours)					
< 10 h	18 (48.6%)	18 (58.1%)	16 (48.5%)	52 (51.5%)	0.68
≥ 10 h	19 (51.4%)	13 (41.9%)	17 (51.5%)	49 (48.5%)	
Sleeping time					
At or before 21:30 h	8 (27.6%)	11 (44%)	11 (35.5%)	30 (35.3%)	0.07
Between 21:30 and 22:30 h	13 (44.8%)	14 (56%)	16 (51.6%)	43 (51.6%)	
After 22:30 h	8 (27.6%)	0	4 (12.9%)	12 (12.9%)	
Bedtime resistance					
Goes to bed at the same time*	1 (1–1)	1 (1–1)	1 (1–1)	1 (1–1)	0.86
Falls asleep in own bed	1 (1–2)	1 (1–2)	1 (1–1)	1 (1–2)	0.98
Falls asleep in other’s bed	1 (1–2)	1 (1–2)	1 (1–2)	1 (1–2)	0.55
Needs a parent in room to sleep	1 (1–2)	1 (1–1)	1 (1–2)	1 (1–1)	0.58
Struggles at bedtime	1 (1–1)	1 (1–1)	1 (1–1)	1 (1–1)	0.15
Afraid of sleeping alone	1 (1–2)	1 (1–2)	1 (1–2)	1 (1–2)	0.26
Total score	8 (6–9.5)	8 (6–10)	8 (7–9)	8 (7–10)	0.67
Sleep onset delay					
Falls asleep in 20 min*	1 (1–1)	1(1–1)	1 (1–1)	1 (1–1)	0.16
Sleep duration					
Sleeps too little	1 (1–1)	1 (1–1)	1 (1–1)	1 (1–1)	0.18
Sleeps the right amount*	1 (1–1)	1 (1–1)	1 (1–1)	1 (1–1)	0.76
Sleeps the same amount daily*	1 (1–1)	1 (1–1)	1 (1–2)	1 (1–2)	0.30
Total score	3 (3–4)	3 (3–4)	3 (3–4)	3 (3–4)	0.60
Sleep anxiety					
Needs a parent in room to sleep	1(1–2)	1(1–1)	1 (1–2)	1 (1–2)	0.30
Afraid of sleeping in the dark	2(1–2)	1(1–2)	1 (1–2)	1 (1–2)	0.15
Afraid of sleeping alone	1 (1–2)	1 (1–2)	1 (1–2)	1 (1–2)	0.64
Trouble sleeping away	1 (1–1)	1 (1–1)	1 (1–1)	1 (1–1)	0.67
Total score	5 (4–7)	5 (4–7)	6 (5–7)	5 (4–7)	0.49
Night wakings					
Moves to another’s bed at night	1 (1–2)	1 (1–2)	1 (1–2)	1 (1–2)	0.24
Wakes once during the night	1 (1–2)	1 (1–2)	1 (1–2)	1 (1–2)	0.99
Awakes more than once	1 (1–1)	1 (1–1)	1 (1–1)	1 (1–1)	0.21
Total score	4 (3–5)	3 (3–4)	3 (3–4)	3.5 (3–5)	0.75
Parasomnias					
Wets the bed at night	1 (1–1)	1 (1–1)	1 (1–1)	1 (1–1)	0.12
Talks during sleep	1 (1–2)	1 (1–1)	1 (1–1)	1 (1–1)	0.78
Restless and moves a lot	1 (1–1)	1 (1–1)	1 (1–2)	1 (1–1)	0.39
Sleepwalks	1 (1–1)	1 (1–1)	1 (1–1)	1 (1–1)	0.11
Grinds teeth during sleep	1 (1–1)	1 (1–1)	1 (1–1)	1 (1–1)	0.56
Awakens screaming or sweating	1 (1–1)	1 (1–1)	1 (1–1)	1 (1–1)	0.16
Alarmed by nightmares	1 (1–1)	1 (1–2)	1 (1–2)	1 (1–2)	0.26
Total score	8 (7–9)	8 (7–9)	8 (7–9)	8 (7–9)	0.84
Sleep-disordered breathing					
Snores loudly	1 (1–1)	1 (1–1)	1 (1–1)	1 (1–1)	0.66
Stops breathing periodically	1 (1–1)	1 (1–1)	1 (1–1)	1 (1–1)	0.32
Snorts and gasps	1 (1–1)	1 (1–1)	1 (1–1)	1 (1–1)	0.11
Total score	3 (3–3)	3 (3–3)	3 (3–3)	3 (3–3)	0.76
Daytime sleepiness					
Wakes by himself or herself*	3 (2–3)	2 (1–3)	2 (1–3)	2 (2–3)	0.00*
Others wake the child	1 (1–1)	1 (1–2)	1 (1–2)	1 (1–2)	0.34
Has a hard time getting out of bed	1 (1–3)	1 (1–2)	2 (1–3)	2 (1–3)	0.18
Takes a long time to become alert	1 (1–2)	1 (1–2)	2 (1–2)	1 (1–2)	0.16
Seems tired at the morning	1 (1–1)	1 (1–1)	1 (1–2)	1(1–1)	0.65
Fall asleep					
Watching TV**	1 (1–2)	0 (0–1)	1 (0–2)	1 (1–2)	0.55
Riding in car**	0 (0–1)	1 (0–2)	1 (0–2)	0 (0–1)	0.86
Total score	11 (10–12)	10 (9–12)	11 (9.5–13)	11 (10–13)	0.71
CSHQ total score	42 (39–46)	41.50 (38–47)	42 (37.5–46)	42 (38–46)	0.99

The frequency of occurrence within the past week: Usually (5-7/week): 3 pt, sometimes (2-4/week): 2 pt, rarely (0-1/week): 1 pt*Reverse items **Not sleepy: 0 pt, very sleepy: 1 pt, Falls asleep: 2 ptPKU: Phenylketonuria, *HPA* Hyperphenylalaninemia, *CSHQ* The Children’s Sleep Habits Questionnaire

In the PKU group, the most recent measured median Phe level was 5.46 mg/dl (3.28–8.81), and the lifelong median was 4.65 mg/dl (3.47–7.04). The last and lifelong median Phe levels were 6.63 mg/dl (IQR: 2.55–9.30) and 4.60 mg/dl (IQR: 3.21–7.09), respectively, for those with clinically significant sleep scores, and 4.79 mg/dl (IQR: 3.53–8.64) and 4.75 mg/dl (IQR: 3.62–7.02), respectively, for those without clinically significant scores. There were no significant differences in last and lifelong median Phe levels between patients with clinically significant and non-significant sleep scores (*p* = 0.21 and *p* = 0.34, respectively). The correlations between phenylalanine levels and BISQ scores are presented in Table 3.

**Table 3:** Correlations of phenylalanine levels and BISQ scores in patients with PKU

Variables	Last Phe	Lifelong Phe	Bedtime resistance	Sleep onset delay	Sleep duration	Sleep anxiety	Night wakings	Parasomnia	Sleep-Disordered breathing	Daytime sleepiness	Total CSHQ score
Last Phe	1										
Lifelong Phe	0.545** (*p* = 0.0)	1									
Bedtime resistance	−0.210 (*p* = 0.21)	−0.053 (*p* = 0.76)	1								
Sleep Onset delay	0.029 (*p* = 0.87)	0.132 (*p* = 0.44)	0.454** (*p* = 0.01)	1							
Sleep duration	0.077 (*p* = 0.65)	0.146 (*p* = 0.39)	0.071 (*p* = 0.68)	0.667** (*p* = 0.0)	1						
Sleep anxiety	−0.347* (*p* = 0.04)	−0.233 (*p* = 0.17)	0.812** (*p* = 0.0)	0.177 (*p* = 0.30)	−0.223 (*p* = 0.19)	1					
Night wakings	−0.161 (*p* = 0.34)	−0.218 (*p* = 0.20)	0.212 (*p* = 0.22)	0.263 (*p* = 0.12)	0.107 (*p* = 0.53)	0.150 (*p* = 0.38)	1				
Parasomnia	0.079 (*p* = 0.64)	0.057 (*p* = 0.74)	0.533** (*p* = 0.0)	0.571** (*p* = 0.0)	0.429** (*p* = 0.01)	0.328 (*p* = 0.05)	0.215 (*p* = 0.20)	1			
Sleep-disordered breathing	0.035 (*p* = 0.84)	−0.166 (*p* = 0.33)	0.127 (*p* = 0.46)	0.350* (*p* = 0.04)	0.418** (*p* = 0.01)	0.101 (*p* = 0.56)	0.397* (*p* = 0.015)	0.378* (*p* = 0.02)	1		
Daytime sleepiness	−0.081 (*p* = 0.63)	−0.199 (*p* = 0.24)	0.118 (*p* = 0.49)	−0.173 (*p* = 0.31)	−0.122 (*p* = 0.48)	0.257 (*p* = 0.14)	0.109 (*p* = 0.53)	−0.027 (*p* = 0.88)	0.110 (*p* = 0.52)	1	
Total CSHQ score	−0.162 (*p* = 0.35)	−0.123 (*p* = 0.48)	0.793** (*p* = 0.0)	0.636** (*p* = 0.0)	0.397* (*p* = 0.02)	0.634** (*p* = 0.0)	0.494** (*p* = 0.0)	0.723** (*p* = 0.0)	0.514** (*p* = 0.00)	0.353* (*p* = 0.04)	1

*Phe* Phenylalanine, *HPA* Hyperphenylalaninemia, *CSHQ* The Children’s Sleep Habits Questionnaire *p<0.05 ** *p*<0.01

For children with HPA, the lifelong and last median Phe levels were 3.1 mg/dl (IQR: 2.55–4.24) and 3.32 mg/dl (IQR: 2.51–3.92), respectively. The last and lifelong median Phe levels were 3.01 mg/dl (IQR: 2.16–3.93) and 2.82 mg/dl (IQR: 2.41–4.07), respectively, for those with clinically significant sleep risk scores, and 3.57 mg/dl (IQR: 3.01–3.76) and 3.54 mg/dl (IQR: 2.81–4.13), respectively, for those without clinically significant scores (*p* < 0.05). The correlations between phenylalanine levels (last and lifelong) and BISQ scores are presented in Supplementary Table 1 . No significant relationships were observed between last and lifelong Phe levels and BISQ scores in patients with HPA (*p* > 0.05).

The relationship between sleep hygiene-related factors (total screen time, the median time for stopping physical activity or food intake, and the median time for turning off screens before bedtime) and clinically significant BISQ scores was evaluated. Among children with HPA, the median time for turning off screens before bedtime was significantly shorter in those with clinically significant BISQ scores (30 min; 0–60) compared to those without clinically significant scores (60 min; 60–60) (*p* = 0.01). Similarly, the median time for stopping food intake was longer in those with clinically significant scores (120 min; 75–125) compared to those without clinically significant scores (45 min; 30–120) (*p* = 0.05). In the control group, the median time for stopping food intake was also significantly longer in those with clinically significant BISQ scores (120 min; 90–180) compared to those without clinically significant scores (60 min; 30–90) (*p* = 0.01). However, among children with PKU, no significant relationship was found between these variables and the presence of clinically significant BISQ scores.

## Discussion

Children with chronic disorders are generally at a higher risk for sleep problems. For children with PKU, this vulnerability may extend beyond the typical challenges of a chronic illness. In this study, we evaluated the sleep characteristics of school-aged children with PKU compared to those with HPA and healthy controls. To the best of our knowledge, this is one of the few studies examining this age group and the first to assess sleep in children diagnosed with HPA. Additionally, evaluating phenylalanine levels in relation to sleep characteristics provides valuable insights into an area that has not been extensively studied.

The prevalence of clinically significant sleep scores in our study, where approximately one in two children had such scores, aligns with findings in similar age groups in our country. A study using the same assessment tool (CSHQ) reported a higher prevalence, with 65.3% of children in both the type 1 diabetes mellitus group—as another chronic disease with predominantly dietary treatment—and the control group having clinically significant scores [24]. It was observed that children in the PKU group were significantly less likely to be awakened by themselves. Apart from this finding, there were no significant differences in other sleep-related characteristics among the PKU, HPA, and control groups. This aligns with findings in the literature, including a study involving 32 children with PKU (mean age: 12 years) and another study evaluating 19 children aged 4–10 years using the same assessment scale as our study, both of which reported no significant differences in sleep characteristics [20, 25]. Interestingly, while increased sleep disturbances have been reported in adults with PKU [19], findings in pediatric populations appear to contrast, showing fewer or no significant deviations from healthy controls in many aspects of sleep. This divergence might reflect differences in disease management, neurodevelopmental factors, or age-related variations in sleep patterns and their underlying biological regulators, such as neurotransmitters and melatonin pathways. The discrepancy observed in the ’wakened by himself/herself’ item may be attributed to the tendency of children with PKU to have later sleeping times. This finding is approaching statistical significance (*p* = 0.07), despite children with PKU having similar total sleep durations compared to their peers. This could potentially stem from disruptions in the melatonin axis or behavioral traits frequently seen in children with chronic illnesses. Furthermore, younger children with chronic conditions often depend heavily on their family members, which might delay the development of independent sleep habits. Further research is necessary to better understand these dynamics [26].

Although the literature suggests a potential association between Phe levels and sleep parameters, this topic remains underexplored, with only one small-scale study, which was mentioned earlier as a study using the same scale and reporting no significant differences, available. That study, which included a limited number of well-controlled cases, evaluated average year and lifetime Phe levels but found no significant relationship between Phe levels and sleep characteristics [25]. Similarly, in our study, no significant relationships were identified between last and lifelong median Phe levels and CSHQ scores, except for an association with sleep anxiety. Interestingly, higher last phenylalanine levels were linked to lower sleep-related anxiety, potentially due to a shift in attention toward other health concerns. Another possibility is that higher phenylalanine levels might increase parental sensitivity toward the child, fostering greater intimacy between the parent and child. This increased parental support, including assistance during the child’s bedtime routine and sleep initiation, could help reduce the child’s sleep anxiety. These interpretations are speculative and highlight the need for further research, particularly with anxiety-specific assessments, to draw more definitive conclusions.

When examining sleep hygiene factors separately for the HPA, PKU, and control groups, distinct patterns emerged. In the HPA group, a shorter median screen-off time before bedtime and a longer median time for stopping food intake were associated with clinically significant BISQ scores. Similarly, in the control group, longer food intake intervals before bedtime were linked to sleep disturbances. Existing recommendations suggest that a healthy, balanced diet promotes good sleep, while consuming large meals close to bedtime may impair sleep quality. Specific snacks, such as those containing carbohydrates, are proposed to support better sleep. However, as our study did not evaluate dietary content, the conclusions regarding its impact on sleep remain limited. Regarding screen time, our findings are consistent with the recommendations, which highlights its negative influence on sleep [27, 28].^.^ Notably, among children with PKU, no significant relationship was identified between these variables and the presence of clinically significant BISQ scores. This observation suggests that different determinants may influence sleep patterns in children with PKU, warranting further investigation.

Gastrointestinal symptoms in children diagnosed with PKU may be potential factors influencing sleep disturbances. This perspective is supported by existing literature and studies highlighting the need to assess sleep problems and gastrointestinal symptoms while addressing significant heterogeneity in management practices [21]. In our study, the relationship between sleep problems and reflux was specifically investigated. However, a broader evaluation of gastrointestinal symptoms was not conducted, as the focus remained solely on reflux. While no significant association was identified between reflux and sleep problems, this finding may be attributed to the limited sample size and the lack of comprehensive gastrointestinal assessment tools. Future research employing standardized and validated methods may provide a deeper understanding of the interplay between gastrointestinal issues and sleep disturbances in children with PKU. Beyond biological factors, many contextual elements influence sleep, including the sleep environment, practices, and psychosocial dynamics [29]. Parents play a crucial role in these psychosocial dynamics. A study conducted at the same institution as our research demonstrated that the mean anxiety symptom scores of mothers of children with PKU were significantly higher compared to nonclinical adult scores reported in the literature [30]. While we did not specifically evaluate the psychological context in our study, this finding suggests that exploring the psychological dimensions and their potential influence on both children and their caregivers may provide valuable insights.

While our research did not reveal significant differences in sleep characteristics among PKU, HPA, and control groups, it highlighted the complexity of factors influencing sleep in these populations. Addressing sleep issues in children with PKU remains crucial due to the developmental and behavioral challenges they face. This underscores the importance of adopting a comprehensive approach to understanding and managing sleep disturbances in this vulnerable group.

This investigation contributes to the existing literature by evaluating sleep hygiene factors, incorporating a distinct HPA group, and analyzing phenylalanine levels in relation to sleep characteristics. Additionally, focusing on a school-aged population provides a unique perspective on this underexplored topic. However, certain limitations must be acknowledged. The reliance on parent-reported data introduces potential reporting bias, and the evaluation of sleep hygiene and gastrointestinal symptoms was not exhaustive. Furthermore, the excellent Phe control observed in our participants may have contributed to the lack of significant sleep problems identified. Individuals with poor metabolic control or late-diagnosed PKU might exhibit more pronounced sleep disturbances, emphasizing the need for future research employing standardized methodologies and more detailed assessments to uncover the mechanisms affecting sleep.

In conclusion, although the findings did not identify significant group differences in sleep characteristics, this should not diminish the importance of understanding the nuanced factors influencing sleep in patients with PKU. Increased awareness of these determinants—spanning biological, behavioral, and environmental domains—is essential. By addressing these complexities through targeted and multidisciplinary interventions, future research can pave the way for improved health outcomes and a better quality of life for children with PKU.

## Supplementary Information

Below is the link to the electronic supplementary material.

ESM 1 (DOCX 19.1 KB)

## Data Availability

No datasets were generated or analysed during the current study.
