# Supplementary material for: Assessing sleep habits in school-aged children with phenylketonuria: a comparative study
Source: Eur J Pediatr. 2025 Mar 5;184(3):225. doi: 10.1007/s00431-025-06048-1 (PMC11882617; doi:10.1007/s00431-025-06048-1)
Supplement: Supplementary file 1 — (DOCX 19.1 KB) [file 431_2025_6048_MOESM1_ESM.docx]

**Supplementary Table 1**: Correlations of Phenylalanine Levels and BISQ Scores in Patients with HPA.

|  |
| --- |

| **Variables** | **Last Phe** | **Lifelong Phe** | **Bedtime Resistance** | **Sleep Onset Delay** | **Sleep Duration** | **Sleep Anxiety** | **Late Sleep Onset** | **Parasomnia** | **Sleep-Disordered Breathing** | **Daytime Sleepiness** | **Total CSHQ Score** |
| --- | --- | --- | --- | --- | --- | --- | --- | --- | --- | --- | --- |
| **Last Phe** | 1 |  |  |  |  |  |  |  |  |  |  |
| **Lifelong Phe** | .708** (p=0.0) | 1 |  |  |  |  |  |  |  |  |  |
| **Bedtime Resistance** | 0.232 (p=0.22) | 0.064 (p=0.74) | 1 |  |  |  |  |  |  |  |  |
| **Sleep Onset Delay** | 0.157 (p=0.41) | 0.087 (p=0.66) | 0.103 (p=0.59) | 1 |  |  |  |  |  |  |  |
| **Sleep Duration** | -0.031 (p=0.87) | -0.185 (p=0.34) | 0.223 (p=0.24) | 0.532** (p=0.0) | 1 |  |  |  |  |  |  |
| **Sleep Anxiety** | -0.136 (p=0.48) | -0.249 (p=0.19) | 0.294 (p=0.12) | -0.158 (p=0.40) | -0.084 (p=0.66) | 1 |  |  |  |  |  |
| **Late Sleep Onset** | -0.212 (p=0.27) | -0.241 (p=0.22) | 0.054 (p=0.78) | -0.086 (p=0.66) | -0.054 (p=0.78) | 0.138 (p=0.48) | 1 |  |  |  |  |
| **Parasomnia** | -0.141 (p=0.46) | -0.242 (p=0.21) | 0.561** (p=0.0) | -0.033 (p=0.86) | 0.131 (p=0.49) | 0.026 (p=0.89) | 0.380* (p=0.04) | 1 |  |  |  |
| **Sleep-Disordered Breathing** | 0.266 (p=0.16) | -0.045 (p=0.82) | -0.049 (p=0.8) | -0.076 (p=0.69) | 0.169 (p=0.37) | 0.072 (p=0.71) | -0.273 (p=0.15) | 0.105 (p=0.58) | 1 |  |  |
| **Daytime Sleepiness** | 0.117 (p=0.555) | 0.348 (p=0.08) | 0.262 (p=0.18) | -0.164 (p=0.41) | -0.153 (p=0.44) | 0.174 (p=0.38) | 0.083 (p=0.68) | -0.031 (p=0.88) | 0.157 (p=0.43) | 1 |  |
| **Total CSHQ Score** | 0.037 (p=0.85) | 0.084 (p=0.68) | 0.805** (p=0.0) | -0.021 (p=0.92) | 0.156 (p=0.44) | 0.244 (p=0.22) | 0.335 (p=0.09) | 0.653** (p=0.0) | 0.087 (p=0.67) | 0.631** (p=0.0) | 1 |

Phe: Phenylalanine, HPA: Hyperphenylalaninemia, CSHQ: The Children’s Sleep Habits Questionnaire

*p<0.05 ** p<0.01
